# Editorial: Omics-based approaches in stroke research

**DOI:** 10.3389/fneur.2024.1472912

**Published:** 2024-08-13

**Authors:** Shubham Misra, Pradeep Kumar

**Affiliations:** ^1^Department of Neurology, Yale University School of Medicine, New Haven, CT, United States; ^2^Clinical Research Unit, All India Institute of Medical Sciences, New Delhi, India

**Keywords:** stroke, omics, biomarker, radiomics, management-healthcare

Stroke remains a leading cause of mortality and disability worldwide, posing substantial challenges to healthcare systems and necessitating innovative research approaches (1). Traditional epidemiological and clinical studies have provided significant insights into stroke risk factors and outcomes (2). However, the advent of omics technologies, including genomics, transcriptomics, proteomics, metabolomics, and radiomics, has revolutionized our ability to understand the molecular underpinnings of stroke (3–5). Omics-based studies have led to the discovery of potential biomarkers that can aid in early detection, risk stratification, and monitoring of stroke patients (6–9). Furthermore, these approaches can uncover novel therapeutic targets, facilitating personalized treatment strategies and the repurposing of existing drugs for stroke management (10).

The *Research Topic* “*Omics-based Approaches in Stroke Research*” in *Frontiers in Neurology* comprises nine articles that leverage omics technologies to advance stroke research. This editorial frames the goals and findings of this research, highlighting their contributions to our understanding of stroke risk, prognosis, and recovery.

Chen et al. used Mendelian randomization (MR) techniques (11) to investigate the causal associations of serum urate (SUA) with stroke risk and prognosis. They found that genetically predicted higher SUA levels increased the risk of any stroke and ischemic stroke (IS) while simultaneously improving post-stroke recovery outcomes. This dual effect was mediated, in part, by systolic and diastolic blood pressures, underscoring the intricate interplay between metabolic and cardiovascular factors in stroke pathophysiology. These findings highlight the importance of considering both the detrimental and protective roles of metabolic factors like SUA in stroke management and rehabilitation strategies. Hu et al. explored the causal relationship between the IgD-CD24-B cell absolute count (IgD-CD24-AC) and IS, along with the potential mediating role of ascorbic acid 2-sulfate (AA2S). Using an MR approach with Genome Wide Association Study data, they found that higher IgD-CD24-AC was associated with an increased IS risk. Additionally, AA2S was found to mediate a small portion of this effect, suggesting its involvement in the pathway linking IgD-CD24-AC and IS. These insights into the immune mechanisms underlying IS highlight potential targets for immune-based therapies in precision medicine. Wu et al. examined the causal relationship between hemoglobin concentration and stroke using a two-sample MR approach. Analyzing data from the UK Biobank, the FinnGen R9, and MEGASTROKE consortia, they found a negative association between hemoglobin levels and stroke risk. Specifically, higher hemoglobin was linked to a lower risk of overall stroke, IS, and cardiogenic stroke. These findings underscore the potential protective role of hemoglobin concentration in stroke prevention and highlight the importance of managing hemoglobin levels in stroke risk reduction.

Zhang et al. investigated the role of peripheral T cells and their receptor repertoire in predicting outcomes of acute spontaneous intracerebral hemorrhage (SICH). Analyzing peripheral blood mononuclear cells from 45 ICH patients compared to healthy controls, they found that ICH was associated with reduced T cell abundance, heightened T cell activation, and altered T cell receptor (TCR) repertoire. Significant correlations between TCR diversity and clinical outcomes suggest that TCR repertoire profiling could serve as a potential biomarker for assessing ICH prognosis and highlight the need for further research into T cell mechanisms in brain injury and repair. Wang et al. explored the prognostic value of the albumin-corrected anion gap (ACAG) in patients with aneurysmal subarachnoid hemorrhage (aSAH). Comparing the predictive efficacy of ACAG and the standard anion gap (AG) in predicting 30-day mortality among 710 aSAH patients, they found that ACAG is positively correlated with mortality and performs better than AG alone in predicting outcomes. This comprehensive prognostic model showed improved predictive accuracy, suggesting that ACAG is a valuable tool for assessing risk and tailoring treatment strategies in aSAH patients. Stańczak et al. investigated whether circulating microRNA (miRNA) profiles could predict the hemorrhagic transformation (HT) risk after thrombolytic treatment in acute IS patients. Analyzing plasma samples from patients who developed HT and those who did not, they identified trends in miRNA expression changes. Notable findings included differential expression patterns that could potentially be used to predict HT risk, although further validation with larger samples is needed. This study highlights the potential of miRNA profiling, combined with additional biomarkers identified using other omics approaches, to enhance prediction models for thrombolysis-associated complications in stroke patients.

Li et al. evaluated the effectiveness of radiomics models derived from non-contrast CT (NCCT) and CT angiography (CTA) images in predicting early hematoma expansion (HE) in patients with SICH. Analyzing data from 182 patients, they created radiomics models based on NCCT and CTA images and found that these models exhibited superior performance compared to the CTA spot sign, a standard clinical marker. These results suggest that radiomics models based on NCCT and CTA are effective for predicting HE and may reduce the need for CTA, thereby lowering patient exposure to radiation and contrast agents. Nie et al. developed a radiomics model based on perivascular adipose tissue (PVAT) surrounding carotid plaques to differentiate symptomatic from asymptomatic plaques. Analyzing data from 203 patients with carotid plaques, they created a radiomics signature (RS) model that demonstrated high diagnostic performance, significantly outperforming traditional models. This RS model based on PVAT is a valuable tool for assessing plaque risk and could enhance risk stratification for carotid atherosclerotic disease. Zhao et al. reviewed the significant advancements and applications of artificial intelligence in the neuroimaging and rehabilitation of IS patients. They highlighted that integrating radiomics with machine learning significantly enhances the predictive accuracy for acute IS outcomes. Radiomics models, utilizing imaging features from diffusion weighted imaging and NCCT, improve prognosis and risk assessment, especially after mechanical thrombectomy.

In summary, the Research Topic “*Omics-based approaches in stroke research*” provides a comprehensive overview of recent advancements in this field. The nine studies included in this topic highlight the potential of omics technologies to elucidate stroke mechanisms, discover new biomarkers, and enhance prognostic models. These findings emphasize the need for continued research to validate and implement these insights in clinical settings. We anticipate that this Research Topic will inspire further studies and contribute to the development of more personalized and effective stroke therapies and management strategies.
